# Effect of Sodium Ascorbate, Grape Seed Extract, and Aloe Vera Application after In-Office Bleaching on the Bond Strength of Enamel: A 3-Year Evaluation

**DOI:** 10.1155/2023/4625818

**Published:** 2023-11-04

**Authors:** Alexandra Mena-Serrano, Eliana Aldás Fierro, Ximena Estrada, Alejandra Boada, Michel Wendlinger, Michael Willian Favoreto, Alessandro D. Loguercio, Alessandra Reis

**Affiliations:** ^1^School of Dentistry, Universidad de Las Américas, Quito 170517, Ecuador; ^2^Department of Restorative Dentistry, School of Dentistry, State University of Ponta Grossa, Ponta Grossa 84030-900, Brazil

## Abstract

**Introduction:**

Dental bleaching is the first choice to improve smile esthetics, but, in some cases, it needs to be associated with resin composite restoration to obtain a satisfactory result. Unfortunately, the bonding of resin-based materials can be impaired due to residual oxygen molecules, which can decrease the durability of the restoration.

**Objectives:**

To evaluate the effect of the antioxidant application on the bond strength of bleached enamel after 24 hr and 3 years of water storage.

**Methods:**

In total, 84 bovine teeth were used in this study. Of these, 77 were bleached with 35% hydrogen peroxide in a single session for three cycles of 15 min. Then, the specimens were divided into groups (*n* = 7 each): control (without bleaching), without antioxidant (WA) use; application of 10% sodium ascorbate (SA) gel, grape seed (GS) extract, and aloe vera (AV). The restorative procedure was performed immediately after bleaching, 7 and 14 days after bleaching. Specimens were sectioned and evaluated using microtensile bond strength (*μ*TBS). Half of the resin-enamel sticks were tested after 24 hr, and the remaining half after 3 years of water storage. *µ*TBS data were analyzed using a three-way analysis of variance, Tukey's test, and Dunnett's test.

**Results:**

The lowest *µ*TBS values were observed when the restoration was performed immediately after bleaching in the AV, GS, and WA groups when compared with the SA group (*p* < 0.005). However, no significant differences were observed among all groups after 3 years of water storage (*p* < 0.001).

**Conclusions:**

SA at 10% was the most effective antioxidant agent for improving the immediate bond strength. However, independent of the antioxidant agent used, the bond strength values were maintained or recovered after 3 years of water storage. SA at 10% could be used to avoid delayed bonding procedures after in-office whitening without compromising bond strength over time. *Clinical Significance*. The use of antioxidants after dental bleaching can be effective in improving the bonding durability of the adhesive restorations.

## 1. Introduction

The number of people dissatisfied with their smiles has been growing in recent years, whether because of tooth color or nonanatomical alterations in enamel, such as hypomineralization defects [1], increasing the demand for esthetic procedures in the dental office among patients [2]. Consequently, tooth bleaching, either at home or in the office, has become an important requirement [3]. This treatment is based on the application of peroxide gels on the enamel surface of vital teeth, where hydrogen peroxide, through its oxidative process, cleaves the bonds of organic molecules (chromophores), thereby providing the tooth with a whiter structure [4].

However, even though tooth bleaching is the first choice to improve smile esthetics, additional restorative procedures may be required to achieve better results [5]. Many studies have reported that hydrogen peroxide can adversely affect the bond strength of resin composites when the bonding procedure is performed immediately after bleaching. This is attributed to the presence of residual peroxide, which interferes with resin attachment and inhibits resin polymerization [6, 7]. Therefore, the waiting period for bonding procedures after bleaching has been reported to vary from 24 hr to 4 weeks [8, 9].

Various techniques, such as the application of antioxidants, have been suggested to manage the compromised bond strength after bleaching. Sodium ascorbate (SA) is the most indicated therapy to restore the reduced bond strength of bleached enamel [10]. Its mechanism of action involves neutralizing free radicals in the organic system by releasing free electrons. However, its efficacy is influenced by different forms, concentrations, and application times [11, 12].

Naturally occurring antioxidants, such as grape seed (GS) extract and aloe vera (AV), have been introduced recently [13]. These products contain oligomeric proanthocyanidin complexes (OPCs) that have free radical scavenging ability, which is 50 times more potent than that of SA [14, 15]. However, controversial results have been reported regarding the efficacy of these alternative antioxidants. Rahman et al. [16] showed that GS and AV were more effective than 10% SA in restoring the bond strength of bleached enamel. Contrarily, Sharafeddin and Farshad [17] showed that all three antioxidant agents had the same effect.

Despite the ability of these antioxidant agents to restore the immediate bond strength of bleached enamel [16, 18, 19], to date, no study has evaluated the effect of the antioxidant application on the longevity of bond strength of bleached enamel.

Therefore, this study aimed to evaluate the immediate and long-term effect (3 years) of 10% SA, GS, and AV application on the microtensile bond strength (*μ*TBS) to the enamel of composite restorations performed immediately, 7, and 14 days after bleaching. The null hypothesis was that the application of 10% SA, GS, and AV would not improve the 24-hr and 3-year bond strength of composite restorations performed immediately and 7 and 14 days after bleaching.

## 2. Materials and Methods

### 2.1. Selection and Teeth Preparation

In total, 84 bovine teeth obtained from a local slaughterhouse were used in this study. The teeth were disinfected in 0.5% chloramine for 7 days and stored in distilled water until use. The crowns were separated from the roots using a low-speed diamond disc (15HC, IsoMet Diamond Wafering Blades, Buehler Ltd, Lake Bluff, IL, USA) under water cooling. The buccal enamel surface of each tooth was ground on wet silicon carbide paper up to grit 800 to create flat surfaces (Norton, Saint-Gobain Peru S.A., Lima, Peru).

### 2.2. Sample Size Calculation

To estimate the sample size, the mean (±standard deviation) bond strength of One Coat Bond SL (25.66 ± 5.6 MPa) to enamel was considered [20]. Using a two-sided test with a power of 0.80 and *α* = 0.05, the minimal sample size required was calculated as five enamel specimens in each group to detect a difference of 9 MPa among the experimental groups. Two extra enamel specimens from each group were added to compensate for specimens potentially discarded during tooth preparation and restorative procedures.

### 2.3. Experimental Design

The present study was designed to evaluate three main factors: (1) antioxidant treatment, subdivided into four levels (without antioxidant (WA), SA 10%, AV, and GS extract); (2) restoration moment at three levels (immediately after bleaching, after 7 days of the bleaching procedure, and after 14 days of the bleaching procedure); and (3) storage time at two levels (immediate and 3 years). These factors were tested for one two-step etch-and-rinse adhesive system (One Coat Bond SL, Coltene/Whaledent AG, Feldwiesenstrasse Altstätten, Switzerland). In total, 84 were randomly divided into 12 groups (*n* = 7) according to antioxidant treatment and restoration moment. For storage time, specimens from the same tooth were randomly tested at 24 hr or 3 years to obtain a research design balanced by tooth dependency.

### 2.4. Antioxidant Gels Preparation

The antioxidant gels were prepared as follows: SA gel was obtained by mixing 0.5 mg SA (98% purity, Sigma-Aldrich Co., St. Louis, MO, USA) and 5 mL glycerol, to improve the viscosity, as well as maintaining the stability of the solution [21], resulting in a translucent solution (pH = 7.8); GS gel, 5 g of GS extract in the form of powder (Puritan's pride, New York, USA) was collected from de capsules and mixed with 5 mL glycerol to obtain a translucent 5% grape seed gel (pH = 7.2); AV gel, the inner clear gel was collected directly from the plant's green leaves (pH = 4.1).

### 2.5. µTBS Evaluation

A single calibrated operator performed the whitening procedure using 35% hydrogen peroxide gel as an in-office product (Whiteness HP, FGM, Joinville, SC, Brazil) in a single session. The bleaching gel was applied to the enamel buccal surface in a single session for three cycles of 15 min, according to the manufacturer's recommendations. After removing the whitening gel with abundant water, antioxidants were applied for 10 min on the dried enamel surface. Finally, the antioxidant was rinsed for 30 s, and the teeth were stored in distilled water for each group. In the immediate bonding group, the restoration process was initiated as soon as the antioxidant was removed.

Another single calibrated operator performed the adhesive protocol according to the manufacturer's instructions. The flattened enamel surface was treated with 35% phosphoric acid (Etchant Gel S, Coltene/Whaledent AG, Feldwiesenstrasse Altstätten, Switzerland) for 30 s, followed by rinsing with water for 20 s, and air-drying until the appearance of chalky white enamel was evident. Immediately after etching, the two-step etch-and-rinse adhesive system (One Coat Bond SL, Coltene/Whaledent AG, Feldwiesenstrasse Altstätten, Switzerland) was applied vigorously for 10 s, gently air-dried for 5 s, and light-cured with an LED unit at a constant intensity of 1,200 W/cm^2^ for 10 s (Bluephase N, Ivoclar Vivadent AG, Bendererstrasse, Schaan, Liechtenstein).

After the bonding procedure, the enamel surfaces were restored with three increments of resin composite (Brilliant NG, Coltène/Whaledent AG, Feldwiesenstrasse Altstätten, Switzerland), each 2 mm in height, and light-cured for 20 s individually with the same LED curing unit. The restored teeth were stored in distilled water at 37°C for 24 hr before testing. Subsequently, the teeth were sectioned with a water-cooled diamond saw (15HC, IsoMet Diamond Wafering Blades, Buehler Ltd, Lake Bluff, IL, USA) at 400 rpm. The composite restoration was performed immediately and 7 and 14 days after bleaching. The obtained bonded sticks were randomly assigned to be tested immediately or after 3 years of storage in distilled water at 37°C.

Each resin-enamel bonded stick with an area of 0.8 mm^2^, measured with a digital caliper (CD-15CPX, Mitutoyo, Kanagawa, Japan), was fixed to a microtensile bond testing jig with cyanoacrylate resin (Loctite Super Glue Gel Control, Henkel Corporation, Connecticut, the United States) and tested in tension at a crosshead speed of 1.0 mm/min using a device operating in microtensile testing mode (ODEME Biotechnology, Brazil). Failure modes were evaluated under a stereomicroscope at 40× magnification and were classified as cohesive, adhesive, or mixed. Additionally, premature failure was evaluated. All sticks for the same tooth with adhesive and mixed failures were averaged for statistical analysis.

### 2.6. Statistical Analysis

Before submitting the data for analysis using the appropriate statistical test, the Shapiro–Wilk test was performed to assess whether the data followed a normal distribution, and Bartlett's test for equality of variances was performed to determine if the assumption of equal variances was valid (data not shown). Thereafter, the following tests were applied: (1) three-way analysis of variance (ANOVA) for antioxidant vs. restoration time vs. storage time and Tukey's post hoc test (*α* = 0.05) to compare the *µ*TBS among all experimental groups; (2) one-way ANOVA and Dunnett's post hoc test (*α* = 0.05) to compare the *µ*TBS obtained in all experimental groups with those of the control group at each time and; (3) Student's *t*-test for dependent variables to compare the *µ*TBS obtained in the control group at each time point (*α* = 0.05).

## 3. Results

The percentages of specimens with premature failure and the frequency of each fracture pattern mode are listed in Table 1. A few premature (3.2% on average) and cohesive (11.6% on average) failures were observed. Most specimens exhibited adhesive or adhesive/mixed failures (Table 1).

Regarding *µ*TBS, the cross-product interaction (antioxidant vs. restoration time vs. storage time) was statistically significant (*p* < 0.001). The lowest *µ*TBS values were observed when the restoration was performed immediately after bleaching in the WA, AV, and GS groups when compared with the SA group (*p* < 0.005). However, a significant increase in *µ*TBS was observed for all protocols after 3 years of water storage (Table 2; *p* < 0.005).

One-way ANOVA showed a statistically significant difference (*p* < 0.005) between the control and experimental groups. The groups in which the restoration was performed immediately after bleaching and without the antioxidant, as well as the AV and GS groups, showed significantly lower values of *µ*TBS when compared with the control group in the immediate restoration period (*p* < 0.005; Table 2). No significant difference was observed when different experimental groups were compared with the control group after 3 years of water storage (*p* > 0.05; Table 2). In addition, no significant differences were observed when the control groups (immediately and after 3 years) were compared (*p*=0.35).

## 4. Discussion

A decrease in the bond strength of enamel typically occurs when the restoration procedure is performed immediately after tooth bleaching [22]. This may be because of the free radicals remaining in the dental substrate after the decomposition of hydrogen peroxide, which interferes with the polymerization of the adhesive layer, leading to a decrease in adhesive resistance [10]. This was reflected by the reduced bond strength values observed in the present study for the WA group in comparison with the control group. Furthermore, the waiting time of 14 days after bleaching and before restoration was enough to achieve bond strength results similar to those observed in the control group, which is consistent with the findings of previous studies [8, 9]. However, this involves postponement of the restorative procedure, thus prolonging treatment time.

In such cases, antioxidant agents have been suggested to improve the bonding properties of bleached enamel. The use of antioxidants in dentistry is not new. Sodium ascorbate is frequently used before the bonding procedure after tooth bleaching [23–26]. Rodríguez-Barragué et al. [27], through a systematic review and meta-analysis of *in vitro* studies, investigated the effects of antioxidants on the bond strength of composite resins to bleached enamel and found that the application of 10% SA for 1 min was effective in increasing the bond strength [27].

Our findings confirmed this statement; when 10% SA was applied immediately before the bonding procedure after bleaching with 35% hydrogen peroxide, the bond strength of the bleached enamel was similar to that of the control group (no bleaching) at both storage times.

The use of plant extracts (GS and AV) as an alternative to SA has been encouraging, as they are more accessible and potent than SA [14]. GS contains 90% polyphenols, the major constituents of which are OPCs, which are polymers of high molecular weight that comprise the monomers flavan-3-ol(*þ*) catechin and (–) epicatechin. OPCs are found in high concentrations in natural sources, such as grapes, extracts of pine bark, hazelnut tree leaves, and lemon tree bark. As a naturally occurring plant metabolite, GS is safe as an antioxidant for various clinical applications [28].

However, in this study, GS and AV were not effective in increasing the immediate bond strength when the restoration was performed immediately after bleaching. GS reacts with free radicals (oxygen) generated by the degradation of hydrogen peroxide, thereby neutralizing them within the enamel in which they are trapped. Free radicals in the physiological control of cell function [29]. The AV leaf contains nonflavonoid polyphenols, which deactivate and disengage free radicals via inert detoxification [30]. AV contains hydroxyl polyphenols and polysaccharides in the parenchymal tissues. These act as reducing agents and donate hydrogen ions to quench nascent oxygen and counteract the consequences of residual oxygen on the tooth surface by bleaching [31].

Therefore, the worst bond strength results for the GS and AV groups in the immediate period were unexpected. A possible explanation for this finding is that the GS and AV concentrations used were not sufficient to completely neutralize the oxygen molecules. The application of GS and AV improved the bond strength when the restorations were performed after 7 days, with the bond strength higher than that observed 7 days after bleaching without the use of any antioxidant. This indicated a synergistic effect of GS/AV and the time to recover the bond strength. Thus, the authors of the present study hypothesized that an increase in the concentration of GS and AV may be capable of improving the immediate bond strength of enamel.

The most important result of the present study is that for all conditions (with or without bleaching and with or without antioxidants), the bond strength to enamel was maintained after 3 years of water storage. Enamel–resin bonds, when produced by etch-and-rinse adhesives, become more stable over time [32]. This occurs because the enamel does not contain organic components that can degrade over time, which may explain the results of the present study [33, 34].

The most intriguing results were obtained in the groups that showed lower bond strength results when the restoration was performed immediately, but their bond strength values recovered after 3 years. This may be because the deleterious effect of oxygen occurring immediately after bleaching dissipates over time. However, because the lower bond strength values observed immediately after the procedure are because of the presence of oxygen, which directly affects the degree of conversion, the recovery of bond strength values after long-term water storage is unexpected.

The authors of the present study hypothesized that delayed polymerization may have led to an improvement in bond strength. It has been well documented that a higher percentage of the degree of conversion is observed immediately after light curing for several polymeric materials [35–37].

However, there is an additional percentage of the degree of conversion that these materials achieve after light curing [38]. This “post-polymerization” occurs because the photocuring is rapid and is stopped by vitrification while a large amount of free resin volume is trapped in the matrix. The adhesive interface undergoes relaxation to reach a more stable state by losing this excess of free volume, which causes “post-shrinkage.” This reduction in volume can induce the frozen-free radicals to move, approaching and possibly reacting with the double bonds of the methacrylate groups, increasing the degree of conversion [39]. This phenomenon could be directly responsible for an increase in the bonding strength over time, as observed by Bittencourt et al. [40]; nonetheless, more studies are necessary to prove this hypothesis.

The null hypothesis of the present study is partially rejected by the findings that only GS and AV did not improve bond strength values of composite restorations performed immediately after bleaching. On the other hand, after 3 years of water storage, there is no significant difference between the antioxidant groups and the control group.

As with all new dental materials, knowing cytotoxicity is important in the field of dentistry when it comes to using these materials. Recent research has shown that when AV was used as an endodontic medication, no significant cytotoxicity was found for the healthy cells. This seems to be associated with the presence of catalase enzyme, which suppresses the generation of free radicals and improves cell efficacy and conservation, as well as hindering lipid peroxidation [41, 42]. SA and GS, also, unlike other antioxidants, did not generate reactive oxygen species, which can cause oxidative damage to cells. In fact, SA can scavenge these reactive oxygen species and prevent their harmful effects [43], while GS, due to the presence of proanthocyanidins, is capable of inducing apoptosis (programmed cell death) in cancer cells while leaving healthy cells intact [44].

While these findings suggest that these natural extracts may have therapeutic applications, it is important to carefully assess their cytotoxicity in the field of dentistry before using them in clinical settings. More research using other methods like apoptosis and cell cycle analysis, described by Pagano et al. [45], is needed to determine the appropriate dosages and applications of these extracts to minimize any potential cytotoxic effects while still providing their potential benefits.

Therefore, if the bond strength to enamel is naturally recovered, it is needless to apply any antioxidants to the dental surface, mainly because this makes the adhesion procedure more complex. However, future clinical studies are needed to evaluate whether applying an antioxidant could improve the clinical performance of adhesive restorations.

## 5. Conclusion

The application of 10% SA immediately after the bleaching procedure was effective in recovering the immediate bond strength of the bleached enamel. Alternative antioxidants, such as GS and AV, only recovered bond strength values after waiting 7 and 14 days for the bonding procedure after bleaching. However, all the groups recovered bond strength values similar to those of the control group after 3 years of water storage.

## Figures and Tables

**Table 1:** Number of specimens (%) according to fracture mode and the premature failure of all experimental groups.

Experimental groups	Restauration moment	Storage time	Fracture pattern		
			C	A/M	PF
Control (without bleaching)	Immediately	24 hr	4 (11.6)	31 (88.4)	0 (0)
		3 years	6 (19.1)	26 (80.9)	0 (0)

After bleaching (without antioxidants)	Immediately	24 hr	3 (8.8)	31 (91.2)	0 (0)
		3 years	3 (9.1)	30 (90.9)	0 (0)
	7 days after bleaching	24 hr	3 (10.9)	25 (89.1)	0 (0)
		3 years	6 (19.1)	28 (74.9)	2 (6.0)
	14 days after bleaching	24 hr	4 (13.4)	33 (86.4)	0 (0)
		3 years	2 (6.0)	29 (90.9)	1 (3.1)

After bleaching + sodium ascorbate 10%	Immediately	24 hr	5 (15.0)	30 (85)	0 (0)
		3 years	3 (11.2)	22 (81.4)	2 (7.4)
	7 days after bleaching	24 hr	9 (30.0)	21 (70.0)	0 (0)
		3 years	3 (6.3)	29 (93.7)	0 (0)
	14 days after bleaching	24 hr	6 (17.4)	29 (82.6)	0 (0)
		3 years	2 (6.6)	27 (84.1)	3 (9.3)

After bleaching+*Aloe vera*	Immediately	24 hr	1 (2.9)	34 (97.1)	0 (0)
		3 years	1 (7.3)	25 (82.4)	3 (10.3)
	7 days after bleaching	24 hr	7 (24.0)	22 (76.0)	0 (0)
		3 years	1 (3.4)	27 (90.0)	2 (6.6)
	14 days after bleaching	24 hr	4 (12.5)	28 (87.5)	0 (0)
		3 years	2 (7.0)	25 (86.0)	2 (7.0)

After bleaching + grape seed extract	Immediately	24 hr	3 (11.3)	27(88.7)	0 (0)
		3 years	2 (6.6)	24 (76.6)	4 (16.8)
	7 days after bleaching	24 hr	9 (32.9)	20 (67.1)	0 (0)
		3 years	0 (0)	30 (93.7)	2 (6.3)
	14 days after bleaching	24 hr	8 (23.4)	27 (76.6)	0 (0)
		3 years	0 (0)	28 (87.5)	4 (12.5)

Abbreviations: C, cohesive fracture mode; A/M, adhesive or mixed fracture mode; PF, premature failure.

**Table 2:** Microtensile bond strength (*µ*TBS) values (means ± standard deviations) of the different experimental groups.

Experimental groups	Restauration moment	Storage time	
		24 hr ^*∗*^	3 years ^*∗*^ ^*∗*^
Control (without bleaching)	Immediately	28.0 ± 2.8	31.4 ± 4.3

After bleaching (without antioxidants)	Immediately	22.5 ± 3.5^d^ ≠	27.9 ± 3.0^b^ =
	7 days after bleaching	23.5 ± 1.3^d^ ≠	30.3 ± 5.9^ab^ =
	14 days after bleaching	32.6 ± 3.2^a^ =	31.0 ± 4.3^ab^ =

After bleaching + sodium ascorbate 10%	Immediately	27.6 ± 2.6^b^ =	28.1 ± 2.7^b^ =
	7 days after bleaching	27.2 ± 2.9^bc^ =	30.0 ± 5.7^ab^ =
	14 days after bleaching	31.6 ± 1.6^ab^ =	31.0 ± 4.3^ab^ =

After bleaching + *Aloe vera*	Immediately	20.1 ± 1.7^d^ ≠	27.8 ± 5.0^b^ =
	7 days after bleaching	27.7 ± 2.3^bc^ =	31.7 ± 1.4^ab^ =
	14 days after bleaching	30.2 ± 4.5^b^ =	31.8 ± 4.2^ab^ =

After bleaching + grape seed extract	Immediately	21.8 ± 1.6^d^ ≠	31.9 ± 4.4^ab^ =
	7 days after bleaching	27.8 ± 1.3^b^ =	34.6 ± 4.4^a^ ≠
	14 days after bleaching	29.6 ± 1.6^b^ =	31.3 ± 5.1^ab^ =

( ^*∗*^) Similar letters mean statistically similar groups (3-way repeated measures ANOVA and Tukey test; *p*=0.0001). The symbol ( = ) means similarity and the symbol (≠) difference compared to the control group for each time (Dunnett's post hoc test, *p* < 0.05). ( ^*∗*^ ^*∗*^) No significant difference was observed when the 24 hr control group was compared with the 3-year control group (*t*-test, *p* < 0.05).

## Data Availability

All the data generated or analyzed during this study are included in the article.
